# Diet and nutrient status of legume consumers in Sweden: a descriptive cross-sectional study

**DOI:** 10.1186/s12937-020-00544-w

**Published:** 2020-04-03

**Authors:** Céline A. Steib, Ingegerd Johansson, Mohammed E. Hefni, Cornelia M. Witthöft

**Affiliations:** 1 Department of Chemistry and Biomedical Sciences, Linnaeus University, 392 31 Kalmar, Sweden; 2 Department of Odontology, Umeå University, 901 87 Umeå, Sweden; 3 Food Industries Department, Faculty of Agriculture, Mansoura University, Mansoura, 355 16 Egypt

**Keywords:** Diet, Dietary patterns, Legume consumption, Fiber, Folate, Riksmaten adults, Sweden

## Abstract

**Background:**

Legumes are nutrient-dense foods and can be an environmentally sustainable alternative to meat consumption. Data on legume intake are scarce and data on legume consumption in Sweden are lacking. This study investigated dietary intake and dietary patterns, together with iron, vitamin D, and folate status, in relation to legume consumption in Sweden.

**Methods:**

Cross-sectional dietary and biomarker data (n 1760) from the 2011 Riksmaten national survey were analyzed. All legume foods (including soy) were identified from 4-day dietary records and ferritin, folate, and vitamin D status in a subgroup (n 280). Participants were classified into non-consumers and quartiles of legume intake. Principal Component Analysis (PCA) was performed to uncover dietary patterns associated with legume intake. Partial Least Square (PLS) regression was used to identify variables associated with variations in legume consumption.

**Results:**

Legumes were consumed by 44% of the population, with mean (SD) intake of 138 (84) g/d in the highest and 11 (5) g/d in the lowest quartiles. Among consumers, 6% reported being vegetarian, compared with 0.9% among non-consumers. Legume consumers drank less alcohol, but had higher intakes of energy, dietary fiber, folate, thiamin, and several minerals, and more often met recommended intake levels for folate and fiber, critical nutrients in Sweden. Biomarker status did not differ with legume intake. PCA revealed multiple loadings on legumes that generally reflected healthier eating habits for legume-consuming women. PLS revealed that vegetarianism was most influential for high legume intake. Other influential variables were high fruit, tea, nut, and seed intakes. High intake of meat, sodas, fast foods, and sweet foods, together with omnivorism, were influential for low legume intake. The associations were similar for men and women.

**Conclusions:**

This study supports dietary recommendations on inclusion of legumes in a healthy diet. Greater focus on assessment of legume intake is necessary to explore the population-wide health effects of legumes as sustainable meat alternatives, and to reinforce national nutritional guidelines.

## Background

*Leguminosae* or legumes, such as fresh and dry beans and peas, lentils, peanuts, soybeans, and lupins, have been cultivated for thousands of years and are part of many traditional diets [1]. Pulses are a sub-type of legumes, defined by the Food and Agriculture Organization (FAO) as exclusively harvested for the dry grain (hence sometimes referred to as ‘grain legumes’). Fatty legumes such as peanuts are harvested for their oil content and ‘fresh’ legumes, such as green beans, are consumed fresh.

Legume grains are a good source of dietary fiber, in particular soluble dietary fiber, and there is a growing body of literature on the health potential of legumes. For example, consumption of pulses has been found to lower fasting blood glucose and insulin, and to be inversely associated with type 2 diabetes incidence [2, 3]. Legume consumption has also been shown to lower serum total and LDL cholesterol [4, 5]. It is often associated with a lower risk of cardio-vascular disease (CVD) [6, 7], although a recent study [8] reported that higher legume intake is associated with increased risk of CVD mortality, but lower cancer risk, in older high-risk subjects. Dietary folate, mainly acquired through beans and lentils, has been found to be negatively associated with excess body weight [9], and potential chemoprotective effects of legumes, particularly on the colon, have been suggested [10, 11].

Legume foods can play a critical role in the transition towards more sustainable diets. Replacing 50% of meat consumption with legumes would reduce the climate impact of the Swedish diet by 20% [12]. For example, 1 kg of soaked and boiled legumes is responsible for 0.3 kg of CO_2_ emissions, whereas 1 kg of prepared beef emits 45.9 kg CO_2_ [13]. However, dietary recommendations to mitigate climate change must provide adequate nutrition. It is predicted that legume replacements would increase intake of dietary fiber and folate, which are critical nutrients consumed below recommendations by most of the Swedish population [14]. It has also been shown that replacement of one serving per day of red meat or processed red meat with legumes is associated with a lower risk of developing metabolic syndrome [15].

The complex composition of legume foods, i.e., rich in both proteins and complex carbohydrates, has caused inconsistencies in their classification. Some classify them as vegetables and others as protein sources [16]. For example, in the Australian Dietary Guidelines, legumes are mentioned in both the “vegetables” and “lean meats and alternatives” groups [17]. Hence, country-specific differences and various culinary uses of legume foods make accurate dietary assessments challenging. Regrettably, these aspects of legumes have been overlooked in assessments in many countries, including Sweden. Characterization of legume consumers is another essential step for increased understanding of the health benefits of legume consumption.

The objectives of the present study were to describe dietary intake of legumes, the pattern of intake, and the iron, vitamin D, and folate status of legume consumers in Sweden.

## Methods

### Study population and dietary assessment methods

National data from the survey *Riksmaten 2010–2011,* collected by the Swedish National Food Agency (NFA), were used in the analysis. A flow chart of this survey is shown in Supplementary Figure S1 (Additional file 1), and a detailed description can be found elsewhere [14]. In brief, between May 2010 and June 2011, 5003 Swedish residents ranging in ag e from 18 to 80 years were invited to take part in the survey. Participation rate was 36%, and was lowest for people with the lowest level of education (14%), immigrants (27%), and men aged 18–30 years (23%) [14]. A total of 1797 individuals reported everything they ate and drank (excluding supplements) for four consecutive days, using an internet-based food diary and a food portion guide. Starting weekday was randomly assigned and recruitment took place during all seasons of the year, to limit the effects of daily and seasonal variations. Data on food intake and on energy and nutrient contents were derived from an internet platform based on information in the food composition database of the NFA. An additional questionnaire was used to collect information on residence, lifestyle, and living conditions, and supplementary dietary information, including dietary regimen and use of any dietary supplement. A sub-group of 1008 individuals were invited to partake in a biomonitoring project and 30% agreed to participate.

The Riksmaten study was approved by the Regional Ethical Review Board of Uppsala (Dnr. 745/2010), and all participants gave informed consent before entering the study.

Individuals with the most extreme energy intakes (lowest and highest 1%) were excluded from the present analysis (*n 22* women < 652 or > 3103 kcal/d; *n 15* men < 859 or > 4076 kcal/d). The final study sample consisted of 1760 individuals with complete dietary records (56% women), of which 280 also donated blood and urine samples. Additionally, high (*n 2*) and low (*n 313*) energy reporters were identified, using the Goldberg equation modified by Black [18]. In sensitivity analysis, the latter were excluded. Education was categorized into elementary school, high school, and university. Smoking was dichotomized into ‘never or former smoker’ and ‘occasional or current smoker’. Body mass index (BMI) was calculated from self-reported weight and height as “weight [kg]/height [m^2^]”. Information on weight and/or height was missing for 121 individuals, so BMI could not be calculated for 7% of subjects. The Riksmaten questionnaire included a question on the type of diet followed by the participants, with listed options being: omnivorous, semi-vegetarian, lacto-vegetarian, lacto-vegetarian occasionally including fish and eggs, ovo-lacto-vegetarian, vegan, and special diet (for food allergy/intolerance, weight loss, or disease treatment).

### Estimation of legume intake

Of a total of 1909 registered food items, 92 items were identified as members of the *Leguminosae* family or meals containing legumes. These 92 items were classified according to the FAO into: (i) pulses (dry beans, dry peas, chick-peas, and lentils), (ii) fresh legumes (e.g., freshly harvested green beans, snap-peas), (iii) peanuts (including peanut butter), (iv) soy products (soy beverages and soy-based vegetarian substitutes), (v) mixed meals with legumes (e.g., ‘pasta salad with chickpeas and beans’ or ‘pita bread with falafel’), and (vi) sprouts (alfalfa and lentil sprouts). Total legume intake was calculated in grams per day for each individual and adjusted for total energy intake using the residual method. Legume consumers were then categorized into quartiles of average daily consumption of total legumes within sex and age category strata, with Q1 having the lowest and Q4 the highest intake. Non-consumers were kept at zero.

### Nutritional biomarkers

Biomarkers of ferritin, erythrocyte and plasma folate, and vitamin D were previously quantified [19]. Serum and erythrocyte folate concentrations below 6.8 nmol/L and 317 nmol/L, respectively, were considered low [20]. Serum ferritin levels < 15 μg/L were considered an indication of abnormal iron status [20], and plasma 25 (OH) D levels < 50 nmol/L as insufficient vitamin D status. All biomarkers were adjusted for age and sex. In sensitivity analysis, vitamin D, plasma ferritin and folate status were controlled, respectively, for vitamin D, iron and folate supplementation. In a second step, multivitamin supplementation was controlled in all three nutrients’ status.

### Statistical methods

#### Descriptive statistics

Socio-demographic characteristics and nutrient intake of the study population were calculated as means (standard deviation (SD)) for quantitative variables and percentages for categorical variables. Ordered logistic regression models were used to determine the effects of socio-demographic characteristics on legume consumption in Table 1. Generalized linear models were used to compare energy and nutrient intakes between non-consumers and consumers at each of the four levels of consumption. Linear contrast post-estimation was used to test for linear trend across categories of consumption in Table 2.

**Table 1:** Characteristics of the population by category of legume consumption

	Non-consumers (n 987)	Consumers (n 773)				
		Q1 (low)	Q2	Q3	Q4 (high)	*P value*
Age, years	47.3 (16.9)	49.4 (16.4)	49.0 (16.4)	49.1 (16.1)	49.4 (16.3)	0.02*
Women, %	54.3	57.9	57.8	57.5	58.1	0.16
Smoker^a^, %	16.0	12.0	14.0	16.4	18.5	0.88
*Education, %*						0.005*
University	40.9	48.2	45.8	49.7	50.8	
Highschool	45.3	39.6	42.7	37.3	37.2	
Elementary school	13.8	12.2	11.5	13.0	12.0	
*BMI, kg/m*^*2 a*^	25.6 (4.4)	25.1 (3.9)	25.5 (4.4)	25.4 (3.8)	25.2 (4.1)	0.25
Non-obese (BMI < 24.9), %	53.6	55.0	50.3	52.5	54.5	
Overweight (25 < BMI < 29.9), %	33.8	33.5	35.2	36.6	35.4	
Obese (BMI > 30), %	12.6	11.5	14.5	10.9	10.1	
Total legume intake, g/day	0.0 (0)	11.3 (5.3)	26.9 (7.5)	52.7 (13.6)	137.7 (83.7)	
Pulses, g/day		12.2 (5.8)	23.2 (10.3)	45.9 (21.0)	93.5 (60.8)	< 0.001*
% consumers		11.7	17.7	35.2	67.0	
Legume vegetables, g/day		12.9 (5.0)	24.1 (7.9)	34.0 (17.7)	40.4 (30.1)	< 0.001*
% consumers		38.6	39.1	32.1	26.7	
Peanuts, g/day		8.3 (4.5)	18.9 (9.1)	22.3 (16.4)	23.0 (19.4)	< 0.001*
% consumers		31.5	16.7	23.3	12.0	
Soy products, g/day		8.7 (1.1)	26.9 (8.8)	42.4 (18.4)	132.7 (127.5)	< 0.001*
% consumers		1.5	5.7	7.8	19.4	
Sprouts, g/day		5.2 (4.4)	3.0 (2.9)	4.0 (2.6)	7.5 (4.9)	0.002*
% consumers		8.1	1.0	2.1	2.6	
Meal with legumes, g/day		12.7(4.3)	27.7 (7.5)	40.5 (16.9)	70.5 (50.9)	< 0.001*
% consumers		13.2	31.2	37.8	36.6	
*Type of diet, %*						< 0.000*
Omnivorous	97.6	95.8	96.1	94.5	82.2	
Vegetarian	0.9	2.0	2.8	3.8	16.7	
Semi-vegetarian	0.7	2.1	1.7	2.2	7.8	
Lacto-ovo vegetarian	0.1		1.1	0.6	2.8	
Lacto-vegetarian with fish and eggs occasionally	0.1			1.1	5.6	
Lacto-vegetarian					0.6	

Consumers are presented in quartiles of energy-adjusted legume intake. Data are expressed as mean (standard deviation) for continuous variables and % for categorical variables. *P < 0.05 (ordinal logistic regression)

^a^Smokers were defined as “current or occasional smokers”. ^b^BMI data missing for 7% of subjects

**Table 2:** Energy and nutrient intake by category of legume consumption

	Non-consumers (n 987)	Quartiles of legume consumption (n 773)				*P value*	*P for trend*^*a*^
		Q1 (low)	Q2	Q3	Q4 (high)		
Total energy intake, kcal/day	1923 (556)	2057** (551)	1973 (536)	2042** (601)	2028* (551)	< 0.001	0.06
Fat, E%	33.9 (6.9)	34.5 (6.2)	33.5 (5.9)	34.4 (5.9)	33.6 (6.4)	0.78	0.50
Carbohydrate, E%	43.3 (7.8)	42.2 (7.3)	44.1 (6.8)	43.2 (7.1)	44.2 (7.1)	0.23	0.06
Dietary fiber, g/day	19.1 (5.3)	19.9 (6.2)	21.0*** (5.2)	22.2*** (6.1)	25.5*** (7.3)	< 0.001	< 0.001
Protein, E%	16.7 (3.4)	16.5 (3.4)	16.7 (2.9)	16.8 (3.1)	16.7 (3.3)	0.67	0.51
Alcohol, g/day	10.3 (13.2)	13.0* (14.2)	9.2 (11.9)	9.8 (12.9)	8.5** (13.2)	0.03	0.001
Total iron, mg/day	10.5 (2.9)	10.6 (2.6)	10.5 (2.4)	10.8 (2.3)	11.6*** (3.0)	< 0.001	< 0.001
Haem iron, mg/day	1.5 (1.5)	1.3 (1.0)	1.2 (0.9)	1.2*** (1.1)	1.1*** (1.4)	< 0.001	< 0.001
Iron from legumes, % of total iron intake	0	1.7*** (1.3)	4.1*** (2.5)	7.2*** (4.6)	13.3*** (7.5)	< 0.001	< 0.001
Folate, μg/day	254 (88)	250 (62)	273*** (97)	274*** (72)	313*** (118)	< 0.001	< 0.001
Potassium, g/day	3.1 (0.6)	3.1 (5.3)	3.2 (5.6)	3.3** (6.1)	3.3*** (6.4)	< 0.001	< 0.001
Magnesium, mg/day	328 (59.6)	338* (64)	344*** (62)	356*** (64)	382*** (94)	< 0.001	< 0.001
Thiamin, mg/day	1.3 (0.3)	1.2 (0.2)	1.3 (0.3)	1.3** (0.3)	1.3*** (0.3)	< 0.001	< 0.001
Zinc, mg/day	11.1 (2.2)	10.8 (2.1)	10.9 (2.0)	11.2 (2.0)	11.1 (2.3)	0.71	0.23
Calcium, mg/day	896.3 (247.6)	874.1 (238.9)	889.4 (245.4)	910.1 (232.4)	894.9 (283.4)	0.81	0.48
Selenium, μg/day	45.8 (16.3)	48.1 (16.4)	46.1 (14.8)	46.6 (14.9)	47.9 (18.2)	0.13	0.41
Vitamin B6, mg/day	2.1 (0.8)	2.1 (0.8)	2.1 (0.9)	2.1 (0.8)	2.1 (0.6)	0.26	0.22
Vitamin D, μg/day	6.8 (4.0)	8.0*** (4.4)	7.4 (4.3)	7.5* (4.4)	7.4 (5.8)	0.01	0.57

Consumers are presented in quartiles of energy-adjusted legume intake. Data expressed as means (SD) adjusted for total energy intake. E% = % of total energy intake

Mean value was significantly different from that of the non-consumer group: * *p* < 0.05; ** *p* < 0.01; *** *p* < 0.001 (generalized linear model). ^a^ Linear contrast post-estimation

#### Deriving dietary patterns

The 1909 registered food items were ordered into food groups adapted from Ax et al. [21]. In brief, 31 food groups were created on the basis of culinary practice and nutrient composition (see Supplementary Table S1 in Additional file 2). Food intake patterns were evaluated using Principal Component analysis (PCA), which reduces large amounts of observations to a number of principal components while maximizing the variance and identifying structures in the data. PCA was performed separately for men and women, as dietary patterns have been shown to differ between sexes in this particular dataset [21]. The adequacy of the data for PCA analysis was evaluated using correlation analysis, Bartlett’s test of sphericity (< 0.001 for both sexes), and the Kaiser-Meyer-Olkin test (0.46 for men; 0.55 for women). The command *pca* in Stata was used to run the analysis. The component loadings were then “rotated” using orthogonal varimax rotation to facilitate interpretation of the data. In order to identify the number of components to be retained, the Kaiser criterion (eigenvalues > 1.0) was first applied. This led to 14 and 12 components being retained for men and women, respectively. Scree plots of eigenvalues (see Supplementary Figure S3 in Additional file 3) were examined and break points were used to reduce the number of components to be retained. Finally, the interpretability of the components obtained was investigated and all components with eigenvalues > 1.25 were retained.

All descriptive statistics and PCA were conducted using STATA version 14.2 (StataCorp LLC, College Station, Texas, USA) with significance level 5%.

#### Partial least square regression

Partial least square (PLS) regression was used to identify predictor variables that determined consumption of legumes. The predictor variables applied to the model were: BMI, age, smoking habits, education, dietary choices (vegetarian or omnivorous), and the 31 food groups used in PCA. One component each was significant for men and women, explaining 16% (R2 19%) and 3% (R2 8%) of variance, respectively. The PLS analysis was performed using SIMCA 15 (Sartrius Stedim Data Analytics AB, Malmö, Sweden).

This study complied with the Strengthening the Reporting of Observational Studies in Epidemiology – nutrition epidemiology (STROBE-nut) guidelines (see STROBE checklist, Additional file 4).

## Results

### Characteristics and nutrient intake of study participants

Among the 1760 individuals ultimately retained, 44% (n 773) consumed legumes, with median intake of 37 g per day (interquartile range: 17–73), contributing to 2% of the individuals’ total energy intake (median), with no significant difference between the sexes. Pulses, legume vegetables, peanuts, and mixed meals with legumes were more frequently consumed than sprouts and soy products. Importantly, the most frequently consumed soy product was a soy drink (consumed by 1% of the total population) that appeared to be non-fortified at the time (not fortified in vitamin D and B12 nor calcium). Details on total intake of general and specific legume food consumption are shown in Supplementary Table S2 in Additional file 2.

Characteristics of non-consumers and legume consumers by their quartile group classification are presented in Table 1. Proportions of women/men, smoking habits, and BMI did not differ between the quartile groups and non-consumers. There was a trend for increased age (*P* = 0.02) and higher education (*P* = 0.01) as legume consumption increased. In addition, there was a trend (*P* < 0.001) towards an increased proportion of vegetarians in consumers (16.7% in Q4) compared with non-consumers (0.9%) (Table 1). There were no vegans in the study sample.

The proportions of energy originating from carbohydrates, proteins, and fats did not differ between the groups (Table 2). However, total energy intake, fiber, several minerals, and folate increased successively across groups Q1-Q4, whereas alcohol intake decreased (Table 2). Of the legume consumers, 23% consumed between 25 and 35 g/d of dietary fiber, compared with 11% for non-consumers (*P* < 0.001). Among the legume consumers, 32% achieved the recommended folate intake of 300 μg/day, compared with 17% for non-consumers (*P* < 0.001). For women of reproductive age (18–44 years), the recommended intake of 400 μg/day was achieved by 6.0% of the legume consumers, compared with 0.4% for non-consumers (*P* < 0.001) (folic acid supplements not included, data not shown). The recommended vitamin D intake (< 10 μg/day) was achieved by 17% of consumers and 22% of non-consumers (*P* < 0.005).

### Food patterns based on principal component analysis

**Fig. 1:**
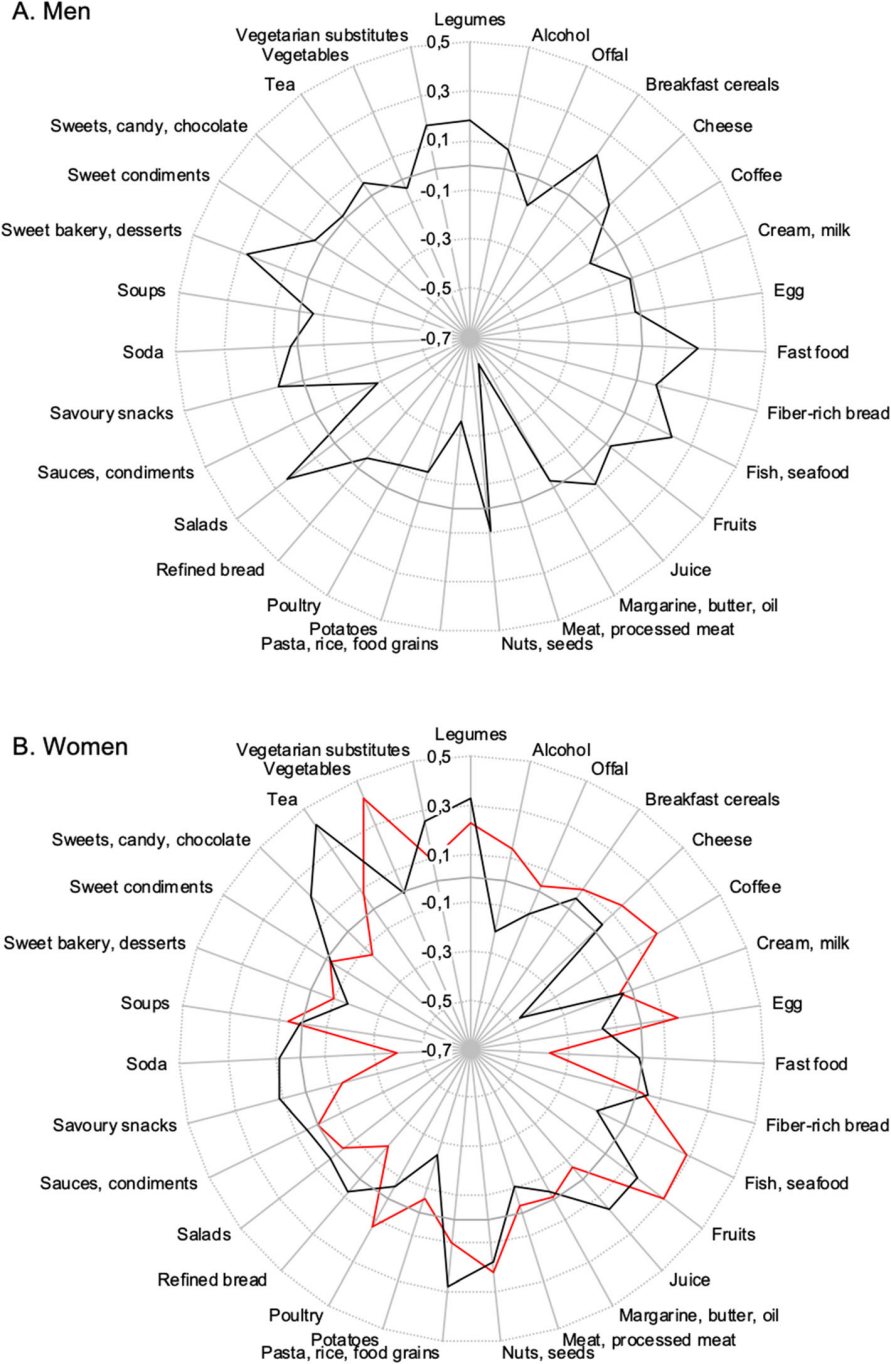
Star diagram illustrating the dietary patterns associated with legume intake for men (**a**: PC5) and women (**b**: PC1 in red, PC2 in black) (only components with loading on legumes > ± 0.15 are shown). Varimax rotated component loadings are presented on the vertical axis, food groups on the radial axis

Six principal components (PC) were retained for men, together explaining 32% of the total variation in the reported diet data. Of these, one component (PC5, explaining 5.1% of variation), and had a varimax rotated loading of 0.18 on the legume food group (Fig. 1, Supplementary Figure S2 in Additional file 2). PC5 loaded positively on fish, breakfast cereals, sweet baked goods, vegetarian substitutes, fast foods, and salads. It loaded negatively on red meat and processed meat products, pasta, rice, sauces, and savory condiments. PC5 was named “Fish and ready-made meals”.

Five PC were retained for women, together explaining 29% of total variation. The first two components loaded positively on legumes. PC1 explained 7% of the variation and had a varimax rotated loading of 0.23 on legumes (Fig. 1, and Supplementary Figure S2, Additional file 2). PC1 was named “Mediterranean” because of its positive loadings on nuts and seeds, vegetables, fruits, and fish. PC2 explained 6% of the variation and had a loading of 0.33 on legumes. PC2 also loaded negatively on most animal-based foods, alcohol, and coffee, but loaded positively on vegetarian substitutes, nuts and seeds, tea, fruits, grains, and candies. It was therefore named “Vegetarian”.

### Partial Least Square analysis

**Fig. 2:**
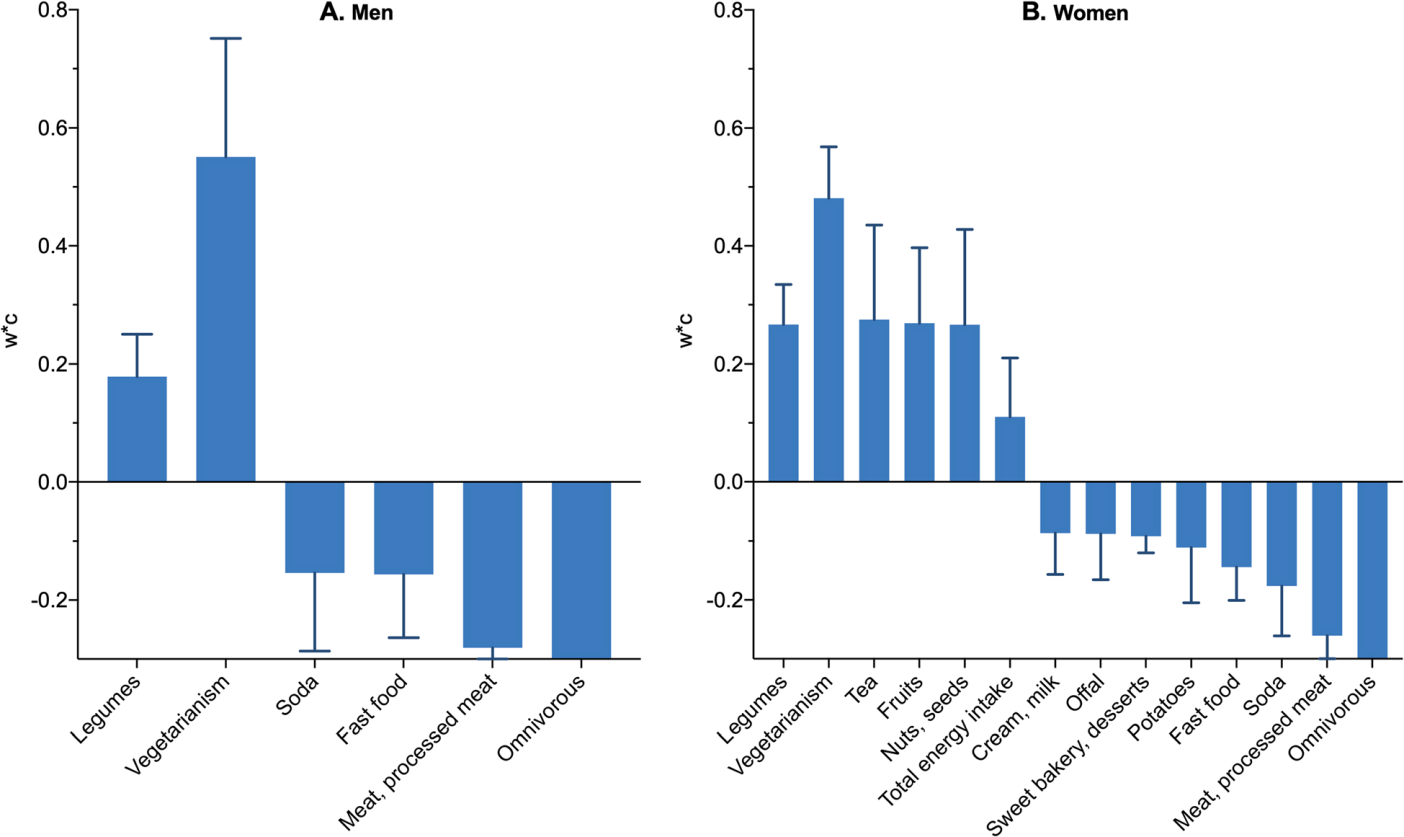
Bar graph of significant w*c coefficients from the PLS model for **a**) men and **b**) women. Error bars show 95% confidence intervals

For women, consumption of tea, fruits, nuts, and seeds had significant positive weights > 0.15 in the PLS model and were more closely associated with legume intake (Fig. 2). Soda, fast foods, and meat showed a negative correlation with legume intake for both sexes, as did dairy products, sweets, offal, and potatoes for women. Choice of diet – omnivorous or vegetarian – strongly predicted legume intake, vegetarianism being associated with higher consumption of legume foods in both men and women.

### Biomarkers of folate, iron, and vitamin D status

There were no differences in serum ferritin, vitamin D, or erythrocyte or plasma folate levels between legume consumers and non-consumers (Table 3), even after sensitivity analysis for supplement use (data not shown).

**Table 3:** Nutrient status of non-legume and legume consumers, including those taking supplements

	Non-consumers	Consumers	*P* value
Plasma folate, nmol/L	15.6 (9.5) (*n* = 136)	17.84 (13.4) (*n* = 141)	0.191
< 6.8 nmol/L, %	3.7	2.8	0.694
Erythrocyte folate, nmol/L	483.3 (116.9) (*n* = 128)	492.7 (122.6) (*n* = 134)	0.630
< 317 nmol/L, %	0.8	2.2	0.336
Ferritin, μg/L	108.5 (88.9) (n = 136)	99.2 (74.7) (n = 141)	0.579
< 15 μg/L, %	6.6	2.1	0.066
Vitamin D 25-OH, nmol/L	58.3 (26.9) (*n* = 137)	58.6 (21.3) (*n* = 139)	0.565
< 50 nmol/L, %	42.0	38.0	0.403

Values expressed as age and sex-adjusted mean (standard deviation) and proportions (%) with low values. Group differences were tested with the Chi-square or Kruskal-Wallis tests

## Discussion

Legumes comprise a variety of products rich in many nutrients, including a comparatively high protein content, making legumes suitable substitutes for meat in transformation to more environmentally sustainable diets. There is a general lack of information on the impact of legume consumption on the overall nutritional value of a legume-containing diet. In this study, we compared nutrient intake, some biomarkers for nutritional status, and the overall dietary and lifestyle pattern of Swedish legume consumers and non-consumers. Some previous studies have characterized legume consumers in other parts of the world [22, 23], but ours is the first to characterize legume consumers in a northern Europe country. We confirmed the hypothesis that legume-rich diets have nutritional advantages and found indications that legume consumers have an overall healthier diet.

To characterize the profile of legume consumers, we used the Swedish cross-sectional, population-based Riksmaten study, where diet intake was recorded over 4 days in adults [14]. We found that only 44% of the study population consumed any type of legume in the four-day period, and that legume consumers were more likely to achieve the recommended intake of folate and fiber, which are critical nutrients in the study population [14]. Another major finding, revealed by multivariate modelling, was that eating habits were healthier among legume consumers than non-consumers, such as less fast-food, sodas, other sweet products, and meat. Although recorded intake of folate and iron increased with increasing legume intake, folate and ferritin status did not vary with legume intake. There are several potential explanations for the lack of association between recorded intake and biomarker status, including imprecise diet recordings, differences in metabolic processing of the nutrients, and the fact that, for practical reasons, blood samples in the Riksmaten cohort were from non-fasting subjects [24].

We found that being a legume consumer, especially being in the highest legume consumption quartile, was associated with increasing prevalence of vegetarianism. This was expected, as legumes are suitable substitutes for meat and as vegetarians and vegans have previously been shown to incorporate more legumes in their diet [25, 26]. Legume consumption also differed with level of education, with high consumers having a higher level of education, which might reflect more nutritional awareness.

Overall, total energy intake increased with legume consumption, but legumes contributed only 3% of total energy intake, confirming previous findings [27]. Despite this, BMI did not differ with legume intake level. This may be partly because legume foods are low energy-density foods, partly because legume consumers may be more health conscious, with higher physical activity, but also because, in the study population, overall consumption was in dishes where other components provide substantial amounts of energy. Lastly, the distribution of total energy intake from the different macro-nutrients is similar between groups of consumers, which may explain similar BMI between groups.

Notably, fiber intake was significantly higher in legume consumers than non-consumers, with twice as many legume consumers achieving the dietary recommendation of 25–35 g/day. As fiber intake is below the recommended level for more than half the Swedish population [14], incorporating more legume foods in the Swedish diet would be beneficial. The major sources of fiber were pulses and legume vegetables, whereas soy drinks did not contribute to fiber intake. Intake of several micronutrients (folate, K, Fe, Mg, thiamin) increased with legume consumption, which reflects the high occurrence of these micronutrients in legumes [4]. Folate in particular is a critical nutrient in Sweden and many other countries, especially for women of reproductive age [19]. In this study, legume consumers, including women of reproductive age, more often achieved the recommended intake of folate. Concerning iron, it is noteworthy that the higher intake of non-haem iron from legumes in the consumer groups did not affect plasma ferritin status, despite their lower intake of haem-iron. Moreover, when excluding iron supplementation, legume consumers more often exhibited adequate ferritin status, which may be explained by their higher intake of vitamin C together with non-haem-iron. Vitamin D intake seemed to decrease with quartiles of legume intake, but more consumers than non-consumers achieved the recommended intake, contradicting findings by Mudryj et al. [22].

Strengths of the present study were the nation-wide, population-based approach employed for participant recruitment and the fact that dietary intake was assessed by repeated recordings, rather than a food frequency questionnaire, and covered several consecutive days, allowing variations in more frequent nutrients in the overall diet to be captured [14]. Still, all methods for recording diet intake suffer from recall bias, with systematic under-reporting being most common and most serious for data inference [28]. To limit the effects of under-reporting, we excluded subjects with the lowest energy intake and those who reported unrealistic energy intake relative to estimated need [18], and we energy-standardized all dietary measures by the residual method [29]. Therefore, despite recording deficits, we believe that the study provides an adequate characterization of legume consumers in Sweden.

Major weaknesses of the study are the comparatively large proportion of non-responders (64%) and the question of representativeness of the target population. A representative sample of the Swedish population was invited to the survey, but the final study group comprised proportionally fewer participants with the lowest education level and men < 30 years of age [14]. Thus, the study group may not be representative of the general Swedish population. However, since there were no major discrepancies in sex, age, and education level between legume categories, we suggest that the data and analyses provide a good general representation of legume consumers in Sweden. Moreover, the data was not modeled for usual dietary intake and therefore only provides estimates.

Another possible limitation of this study is the aggregation of food items in general and legume items in particular, and the possibility to compare with other studies. Definitions for food aggregation differ between studies, which affects the variation reported in dietary patterns [30]. Here we followed the classifications described in previous Swedish studies [21], which allows precise intra-regional comparisons but may limit comparisons with international data. Specifically, classification of legumes differs widely between studies, due to various definitions of the term “legumes” and limitations in the underlying diet assessments. In the present study, all legume-containing foods were identified and classified from the large Swedish national food composition database [14]. This resulted in a detailed description of total legume consumption that is not fully in concert with that in most published studies. For example, Becerra-Tomás et al. [3] define total legume consumption as the sum of lentils, chickpeas, fresh peas, and dry beans, therefore omitting soy products, peanuts, and other legume foods. In the US NHANES cohort, assessment of legume intake included pulses and peanuts, but not soy [31]. Consequently, care is needed when relating our results to those of other studies, e.g., describing consumption and associations with health. As an example, the proportion of pulse consumers was similar in our Swedish study population and a Canadian population, but the types of pulses consumed and portion sizes differed [22]. In Sweden, the most frequently consumed legume foods are legumes incorporated in mixed dishes, freshly harvested green peas and beans, and peanuts. The most frequently consumed pulse food is peas (*Pisum sativum*), in the form of the traditional Swedish split pea soup. In Canada, the most frequently consumed pulse is mung beans, while pinto beans and navy beans are the most popular pulses in the US [22].

## Conclusions

Overall, our results suggest that legume consumption is part of a healthy eating pattern and adequate nutrient intake, but legume consumption in Sweden tends to be relatively low. The insights gained in this study can be of assistance to public health officials in setting nutritional guidelines. Future research should examine the health effects of legume consumption in a longitudinal, population-based cohort.

## Supplementary information

**Additional file 1: Figure S1.** Flow chart of study outline. The analysis were performed on the datasets highlighted in blue, which were provided by Livsmedelverket.se. *For folate analysis, folate and multivitamin takers were excluded. For vitamin D, only participants taking vitamin D were excluded. For iron, participants taking iron supplements were excluded in the first instance, and then participants taking multivitamins (that might contain iron). Grey boxes indicate sensitivity analyses.

**Additional file 2: Table S1.** Description of the food groups used for Principal Component Analysis (PCA). **Table S2.** Frequency of consumption by types of legumes and average intake (g/d) ± SD of consumers, energy-adjusted. *Meals with legumes incorporated. **Figure S2.** Heat map of varimax rotated loadings for the selected principal components loading positively (> 0.15) on legumes. Loadings ± > |0.15| are presented in bold. The color gradient indicates most negative loadings in red and most positive loadings in green. White indicates loading close to 0, meaning that the food group is not representative of the component.

**Additional file 3: Figure S3.** Scree plots of eigenvalues of unrotated factors (A. men, B. women). The red reference line indicates the Kaiser criterion of eigenvalue equal to 1.

**Additional file 4.** STROBE-nut checklist.

## Data Availability

The data supporting the findings of this study are available from Livsmedelverket (https://www.livsmedelsverket.se). However, restrictions apply to the availability of these data, which were used under license in the present study and are not publicly available. Some data are available from the authors upon reasonable request and with the permission of Livsmedelverket.

## Abbreviations

NFA: National Food Agency
CVD: cardio-vascular disease
FAO: Food and Agriculture Organization
PCA: Principal Component Analysis
PLS: Partial Least Square
STROBE-nut: Strengthening the Reporting of Observational Studies in Epidemiology – nutrition epidemiology
