# Supplementary figures and images for: Diet and nutrient status of legume consumers in Sweden: a descriptive cross-sectional study
Source: Nutr J. 2020 Apr 3;19:27. doi: 10.1186/s12937-020-00544-w (PMC7126360; doi:10.1186/s12937-020-00544-w)

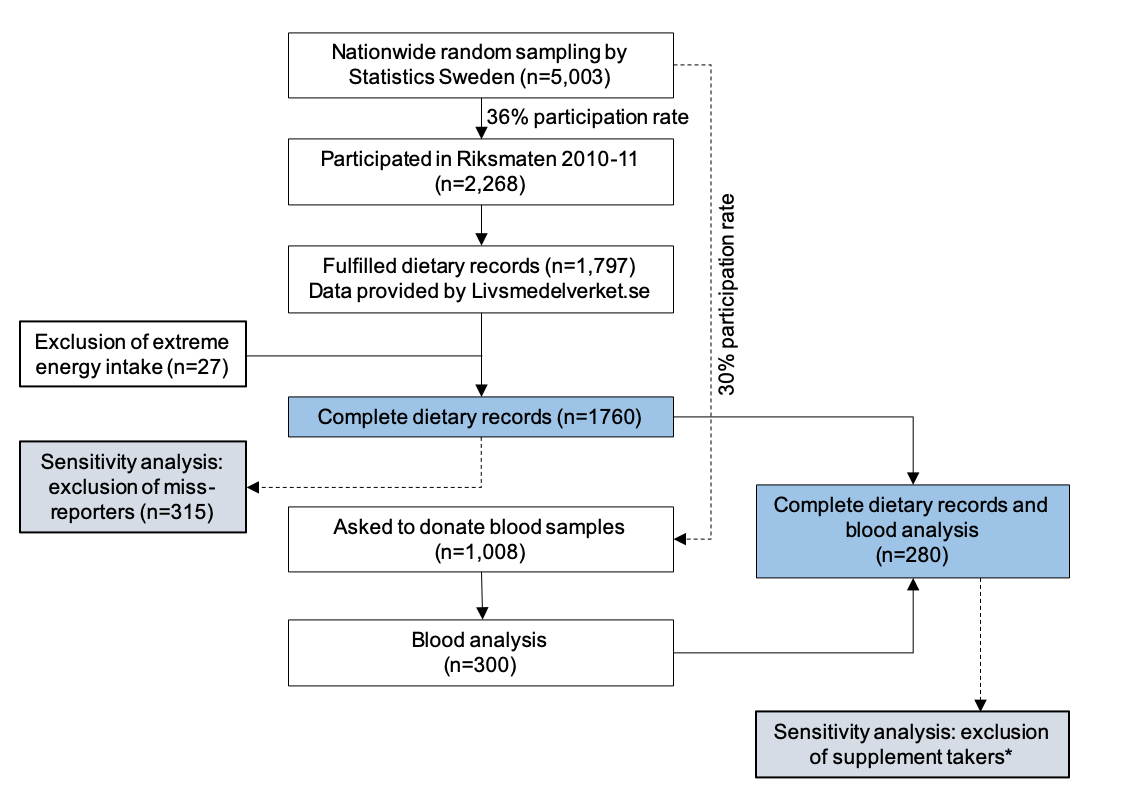

Supplement: Supplementary file 1 — Additional file 1: Figure S1. Flow chart of study outline. The analysis were performed on the datasets highlighted in blue, which were provided by Livsmedelverket.se. *For folate analysis, folate and multivitamin takers were excluded. For vitamin D, only participants taking vitamin D were excluded. For iron, participants taking iron supplements were excluded in the first instance, and then participants taking multivitamins (that might contain iron). Grey boxes indicate sensitivity analyses. [file 12937_2020_544_MOESM1_ESM.png]

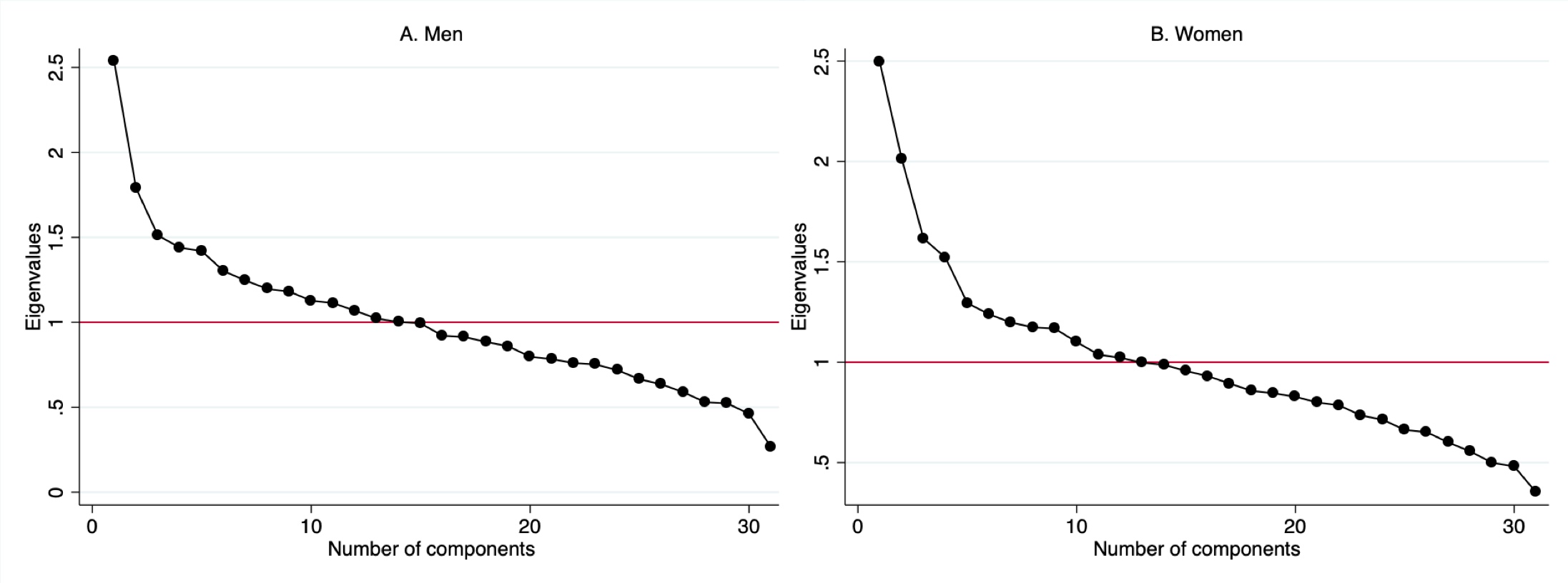

Supplement: Supplementary file 3 — Additional file 3: Figure S3. Scree plots of eigenvalues of unrotated factors (A. men, B. women). The red reference line indicates the Kaiser criterion of eigenvalue equal to 1. [file 12937_2020_544_MOESM3_ESM.png]
